# Genome-Wide Screening of the MYB Genes in *Coptis chinensis* and Their Roles in Growth, Development, and Heavy Metal Resistance

**DOI:** 10.3390/genes16050476

**Published:** 2025-04-23

**Authors:** Yang Yang, Jingmao You, Xuebo Hu

**Affiliations:** 1 Institute for Medicinal Plants, College of Plant Science and Technology, Huazhong Agricultural University, Wuhan 430070, China; life333@webmail.hzau.edu.cn; 2 Innovation Academy of International Traditional Chinese Medicinal Materials, Huazhong Agricultural University, Wuhan 430070, China; 3 Institute of Chinese Herbal Medicines, Hubei Academy of Agricultural Sciences, Enshi 445000, China; jingmaoyou@126.com

**Keywords:** *Coptis chinensis*, CcMYB, genome-wide identification, biosynthesis, cadmium stress, qRT-PCR

## Abstract

**Background:** *Coptis chinensis* is a traditional medicinal plant rich in bioactive compounds like berberine, known for its antibacterial, anti-inflammatory, and antioxidant properties. This study aims to analyze the MYB transcription factor family in *C. chinensis* to better understand their roles in plant growth, development, metabolism, and stress responses. **Methods**: We employed bioinformatics to conduct a genome-wide identification of MYB genes in *C. chinensis*, followed by analyses of physicochemical properties, phylogenetic relationships, gene structures, chromosomal localization, conserved motifs, cis-acting elements, and expression patterns. Results were validated using qRT-PCR. **Results**: A total of 129 CcMYB genes were identified across nine chromosomes. Phylogenetic analysis categorized these genes into 19 subgroups, notably highlighting the S6 subgroup, which lacks counterparts in Arabidopsis. Comparative genomics revealed segmental duplication among gene pairs. Transcriptomic analysis indicated that *CcMYB21*, *CcMYB40*, *CcMYB105*, and *CcMYB116* had high expression levels in stems. Importantly, *CcMYB94* expression significantly increased under cadmium stress, suggesting its role in stress regulation. **Conclusions**: This study offers a comprehensive analysis of the MYB gene family in *C. chinensis*, underscoring the significance of MYB transcription factors in enhancing the plant’s medicinal value and stress tolerance, particularly against cadmium exposure. These insights pave the way for further exploration of specific MYB genes to improve stress resilience in *C. chinensis*.

## 1. Introduction

*C*. *chinensis* is a traditional medicinal herb primarily distributed in the mountainous regions of China, Japan, and South Korea, commonly used in the treatment of heat-clearing, detoxification, anti-inflammatory, and analgesic conditions [1,2,3]. *C. chinensis* belongs to the Ranunculaceae family and the genus *Coptis* and is a perennial herbaceous plant. Coptidis rhizome (CR), known as Huanglian in Chinese, is derived from the rhizomes of *C*. *chinensis* and has been a cornerstone of traditional Chinese medicine for millennia, featured in formulas like Sanhuang-Xiexin-Tang and Gegen-Qinlian-Tang for its health benefits [4,5]. Studies have shown that *C*. *chinensis* is rich in bioactive compounds, such as berberine, which exhibit significant pharmacological effects, including antibacterial, anti-inflammatory, and hypoglycemic activities, thus highlighting its considerable medicinal value [6]. In recent years, research on *C. chinensis* has expanded beyond its traditional therapeutic functions to explore its potential applications in areas such as cancer treatment, antioxidation, and the modulation of the gut microbiome, further enhancing its scientific value in modern medicine [7].

The MYB transcription factor family is one of the most widely distributed and functionally powerful transcription factor families in plants, playing a key role in plant growth, development, metabolism, and responses to environmental stress [8]. The first MYB transcription factor in plants, *ZmMYBC1*, was cloned from maize in 1987, and studies have shown that *ZmMYBC1* primarily participates in anthocyanin synthesis in maize [9]. The core structure of MYB transcription factors is the highly conserved MYB DNA-binding domain, typically composed of one to four imperfect repeat units, each consisting of 50–53 amino acids. Each repeat adopts a helix-turn-helix (HTH) structure, enabling MYB proteins to precisely bind to specific DNA sites, thereby regulating the expression of target genes [8,10]. Based on the number of repeat units in the DNA-binding domain, MYB transcription factors are classified into four major types: 1R-MYB, R2R3-MYB, 3R-MYB, and 4R-MYB [11]. Among these, R2R3-MYB is the most common and extensively studied type, playing a significant role in regulating plant metabolism, development, and stress responses [12]. For example, in *Fagopyrum esculentum*, the overexpression of *FeR2R3-MYB* may enhance antioxidant pathways responding to drought stress and regulate the biosynthesis of anthocyanins [13]. In *Pyrus bretschneideri*, a novel *R2R3-MYB* transcription factor, *PbMYB1L*, enhances cold tolerance and anthocyanin accumulation in transgenic *Arabidopsis* by regulating the expression of genes associated with cold response pathways and anthocyanin biosynthesis [14]. The R2R3-MYB transcription factor *BpMYB1* from *Broussonetia papyrifera* interacts with the DELLA protein BpGAI1, which facilitates the phytoremediation of Cd-contaminated soil [15]. In general, the comprehensive study of the MYB gene family in plants, especially medicinal plants, has certain research value.

Furthermore, MYB transcription factors play a crucial role in regulating the synthesis of secondary metabolites in plants, particularly in the biosynthesis of flavonoids and alkaloids [16]. In the medicinal plant *Scutellaria baicalensis*, *SbMYB3* directly activates the expression of *SbFNSII-2*, promoting the synthesis of root-specific flavonoids [17]. Integrating transcriptomics, metabolomics, biochemical, and genetic analyses have revealed that MYB transcription factors such as *CsMYB8*, *CsMYB85*, and *CsMYB9* in *Camellia sinensis* regulate the biosynthesis of flavonoids, caffeine, theanine, carotenoids, volatiles, lignin, and indole compounds [18]. In *P. bretschneideri,* the R2R3-MYB transcription factor *PbMYB5*-like positively regulates the biosynthesis of phenylalanine-related metabolites [19]. Additionally, in important crops, MYB transcription factors play key roles. For example, in *Zea mays*, *ZmMYB8*, *ZmMYB31*, and *ZmMYB39* are involved in regulating the expression of genes related to lignin synthesis, thereby influencing the synthesis of lignin in the stalks [20]. In *Oryza sativa*, the expression of *OsMYB14* in leaves and roots has been shown to regulate plant height by controlling hormone metabolism [21]. With the increasing availability of plant genomes, research on plant MYB transcription factors has advanced significantly. However, the functions of MYB transcription factors in medicinal plants and the specific mechanisms of their involvement in metabolic regulation still require further investigation.

Here, we identified all the members of the MYB gene family in *C. chinensis* and conducted a detailed analysis of their physicochemical properties, phylogenetic relationships, chromosomal locations, and gene structures. Using transcriptome data, we analyzed the expression patterns of MYB genes in different tissues and identified MYB genes that may be involved in the biosynthesis pathway of berberine. The expression levels of these genes were further validated by qRT-PCR. Additionally, by analyzing metal stress data, we identified key MYB transcription factors involved in heavy metal stress response. This study provides in-depth insights into the MYB gene family of the medicinal plant *C. chinensis*, shedding light on the key *MYB* genes that influence tissue development and potentially participate in heavy metal stress response.

## 2. Materials and Methods

### 2.1. Identification and Sequence Analysis of CcMYB Sequences

The genome sequence and annotation files of *C. chinensis* were downloaded from the NCBI under the BioProject accession number PRJNA649082. The MYB protein sequences of *A. thaliana* were obtained from PlantTFDB (https://planttfdb.gao-lab.org/) (accessed on 5 November 2024) and used to align with the protein sequences of the *C. chinensis* genome. The Hidden Markov Model (HMM) file corresponding to the MYB domain (PF00249) was retrieved from the Pfam database. Subsequently, redundant transcripts were removed from the candidate genes, and the remaining sequences were submitted to the NCBI Conserved Structural Domain Database (NCBI-CDD, https://www.ncbi.nlm.nih.gov/cdd/) (accessed on 29 November 2024) and SMART (https://smart.embl-heidelberg.de/) (accessed on 29 November 2024) databases to confirm protein family domains.

The physicochemical properties of MYB genes in *C. chinensis* were predicted and analyzed using the ExPASy [22] (http://web.expasy.org/protparam/) (accessed on 2 December 2024). MEME [23] (http://meme-suite.org/tools/meme) (accessed on 6 December 2024) was employed to identify motif structures within the proteins, and the genomic annotation files were utilized to determine the gene locations. Subsequently, the gene structures and motif information of MYB genes were visualized using TBtools (v2.156) [24,25]. The upstream 2000 bp sequences of *CcMYB* genes were extracted from the *C. chinensis* genome and analyzed as promoter sequences using PlantCARE (http://bioinformatics.psb.ugent.be/webtools/plantcare/html/) (accessed on 10 December 2024).

### 2.2. Phylogenetic Analysis

The MYB protein sequences of *C. chinensis* and *A. thaliana* were aligned using the software ClustalX (v1.83). The alignment results were then imported into MEGA 7 [26] to construct a phylogenetic tree using the Neighbor-Joining method, with bootstrap analysis set to 1000 replicates and other parameters maintained at their default values.

### 2.3. Chromosomal Localization and Synteny Analysis of CcMYB

In this study, we utilized the genome annotation data of *C. chinensis* to map the chromosomal positions of *CcMYB* genes, employing the TBtools (v12.156) for gene localization and analysis. The homologous relationships among chromosomes were calculated using the MCScanX (v1.0) [27], and the information of homologous gene pairs was organized and visualized using the advanced Circos tool V2.0 to display the duplication events of *CcMYB* genes. Additionally, the KaKs_Calculator 3.0 [28] software was utilized to compute the Ka/Ks.

### 2.4. Expression Analysis of CcMYB Genes

The transcriptome data for cadmium stress were downloaded from the China National Genomics Data Center (CNCB), with the accession number CRA013690. Each control group (CK) and cadmium treatment group included three biological replicates. The cadmium-contaminated and normal soils were collected from contaminated and non-contaminated farmlands, with cadmium concentrations of 0.73 ± 0.015 mg kg^−1^ and 0.26 ± 0.01 mg kg^−1^, respectively. The transcriptome data of the roots, stems, leaves, and flowers of *C. chinensis* were downloaded from the NCBI database, with the accession number PRJNA649082, and each tissue includes three biological replicates.

For subsequent analysis, the downloaded FASTQ files were first quality controlled using FastQC (v0.12.1) [29] to assess the quality of each sample, and low-quality sequences and adapters were trimmed using Trimmomatic (v0.40) [30]. The processed sequences were then aligned to the *C. chinensis* genome using HISAT2 (v2.2.1) [31]. The genome of *C. chinensis* was obtained from NCBI (BioProject accession number: PRJNA662860). Gene-level counting of the aligned reads was performed using featureCounts (v2.0.8) [32]. To eliminate differences in sequencing depth and gene length across samples, FPKM (Fragments Per Kilobase of transcript per Million mapped reads) normalization was applied to the raw count data [33]. To quantify the expression changes between different conditions, log2 fold-change (log2FC) was calculated for each gene. Differential expression analysis was performed using edgeR (v3.20) [34], and all expression data were analyzed in the R programming environment. Transcriptome trend analysis was performed using Mfuzz (v2.66.0) [35].

### 2.5. qRT-PCR Procedures

To assess the reliability and accuracy of the transcriptome data and further explore the expression patterns of *CcMYB* genes, 9 *CcMYB* genes were randomly selected for qRT-PCR analysis. Fresh samples of roots, stems, leaves, and flowers of *C. chinensis* were collected from Enshi, Hubei, China, immediately placed in liquid nitrogen, and stored at −80 °C for RNA extraction. RNA extraction was performed following previously described methods, and a standard amount of RNA was reverse transcribed into complementary DNA (cDNA) using a reverse transcription kit (TAKARA, Beijing, China). Three biological replicates were used for each experimental condition, and each measurement was repeated three times to ensure reproducibility and minimize experimental errors. The expression levels of target genes were calculated using the 2^−ΔΔCT^ method [36], with each measurement repeated three times. The primers used in this experiment are listed in Table S1.

## 3. Results

### 3.1. Identification of MYB in C. chinensis

**Figure 1:**
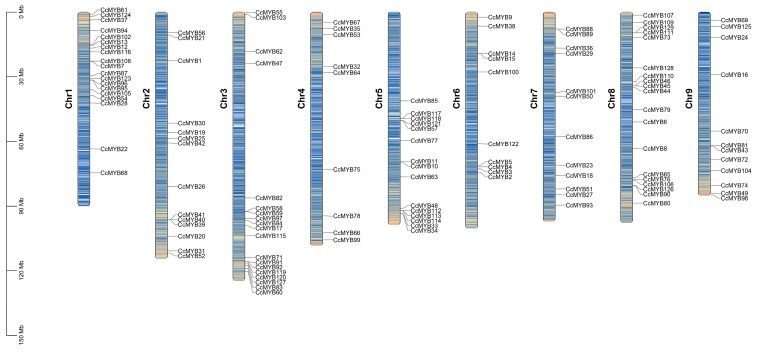
The location of MYB family genes on 9 chromosomes of *C. chinensis* genome; *CcMYB* genes are represented by black font. Different colors represent different gene densities: blue indicates low density, while red indicates high density (divided using a 1Mb window).

Through a comprehensive analysis of the genome data of *C. chinensis*, 129 potential CcMYB gene family members were identified (Table S2), and they were distributed in different degrees on nine chromosomes (Figure 1). Utilizing the available sequence data, an in-depth assessment was conducted to evaluate various characteristics of these genes, including their coding sequences (CDSs), protein sequence lengths, molecular weights (MWs), isoelectric points (pIs), and predicted subcellular localization. The results revealed that the deduced protein lengths of the CcMYBs ranged from 68 amino acids (aa) for *CcMYB104* to 1940 aa for CcMYB6. The molecular weights of these proteins varied significantly, with the largest being 214.24 kDa for *CcMYB6* and the smallest 7.88 kDa for *CcMYB104*. Additionally, based on preliminary predictions from the software, 113 CcMYB proteins are localized in the nucleus, 8 in the cytoplasm, 7 in the chloroplasts, and 1 in the mitochondrion (Table S2).

### 3.2. Phylogenetic Analysis of CcMYB Gene Family

**Figure 2:**
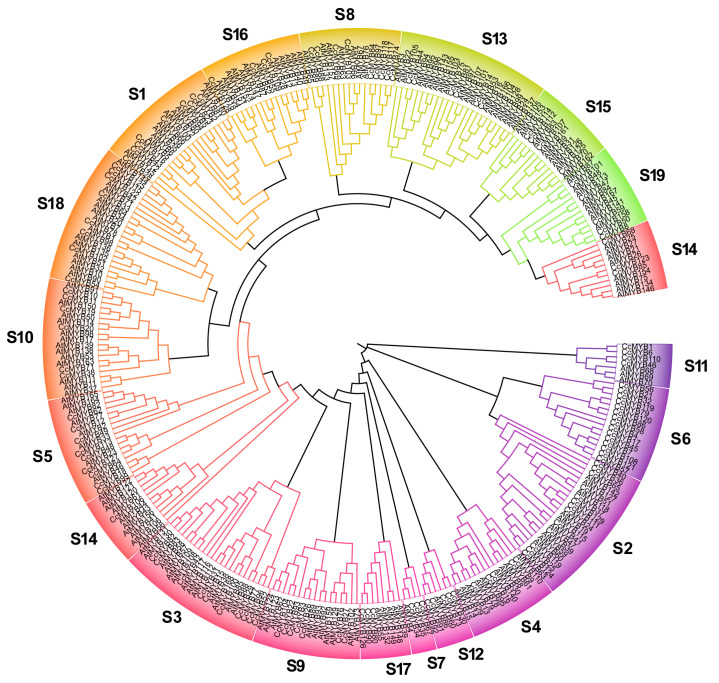
The phylogenetic tree constructed based on the 129 *CcMYB* genes of *C. chinensis* and 168 *AtMYB* genes of *A. thaliana*, with different colors representing different subfamilies.

Phylogenetic analysis of the CcMYB gene family was conducted by identifying 129 MYB genes in *C. chinensis*, phylogenetic tree was constructed, incorporating 168 AtMYB genes from *A. thaliana* for comparative analysis. Based on the phylogenetics of *MYB* genes in *A. thaliana*, the *MYB* genes in *C. chinensis* were broadly categorized into 19 subfamilies (Figure 2). The number of R2R3MYB was 83, belonging to 16 subgroups. The S6 subfamily contained the highest number of *CcMYB* genes (14), while the S11 and S16 subfamilies contained the fewest (4). Compared to *A. thaliana*, we found that in the S6 subfamily, there are only the *CcMYB* genes, but no *AtMYB* gene, which suggests that the function of CcMYB may be different from that of AtMYB. The number of CcMYB genes within each subfamily exhibited significant variation, suggesting that the *C. chinensis* MYB gene family may have undergone functional diversification during its evolutionary process.

### 3.3. Exon/Intron Organization and Motif Composition Analysis of CcMYB Genes

**Figure 3:**
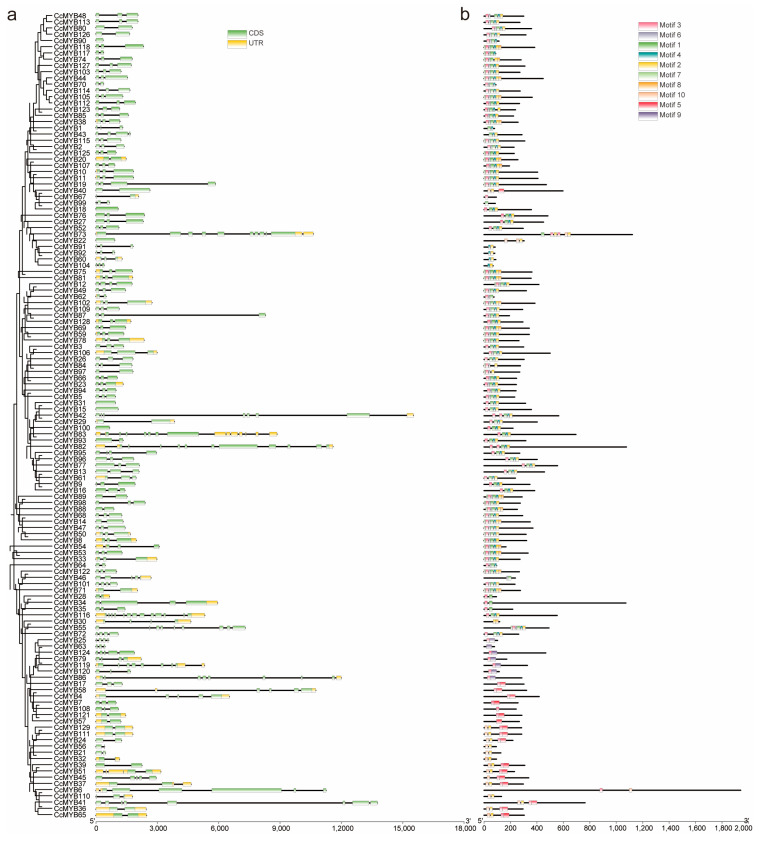
Structural characteristics and conserved motifs of the *CcMYB* genes. (**a**) Phylogenetic relationships and the gene structures of the CcMYB gene. Coding sequence (CDS) regions are represented by green rectangles, untranslated regions (UTRs) are represented by yellow rectangles, and introns are represented by black lines. (**b**) Conserved protein motifs of the *CcMYB* genes.

To provide robust support for phylogenetic analyses, the predicted coding sequences (CDSs) of all identified MYB genes in *C. chinensis* were compared. The exon–intron structures of these genes exhibited considerable variability, with significant differences observed in both the number of exons and sequence lengths across individual genes (Figure 3a). Some members, such as *CcMYB32*, *CcMYB100*, and *CcMYB90*, contained only a single exon, while *CcMYB116* possessed the highest number of exons (12) among all family members. Furthermore, the clustering results from the evolutionary tree revealed that genes within the same branch typically shared similar structural configurations, as exemplified by *CcMYB36* and *CcMYB65*.

In addition to analyzing the structural characteristics of the genes, the predicted amino acid sequences of the MYB proteins were analyzed using the MEME suite to identify conserved motifs and sequence patterns within the protein family. The results revealed variations in motif compositions among different genes, while certain motifs, such as motif1, motif3, and motif6, were present in nearly all family members, representing conserved structural elements of the MYB gene family. Notably, the protein structures of genes within the same evolutionary branch exhibited high conservation (Figure 3b). Previous studies have revealed that genes containing specific motifs may have undergone significant functional evolution. Therefore, investigating the roles of non-conserved motif sequences is of importance.

### 3.4. Cis-Acting Elements Analysis of CcMYB Genes

**Figure 4:**
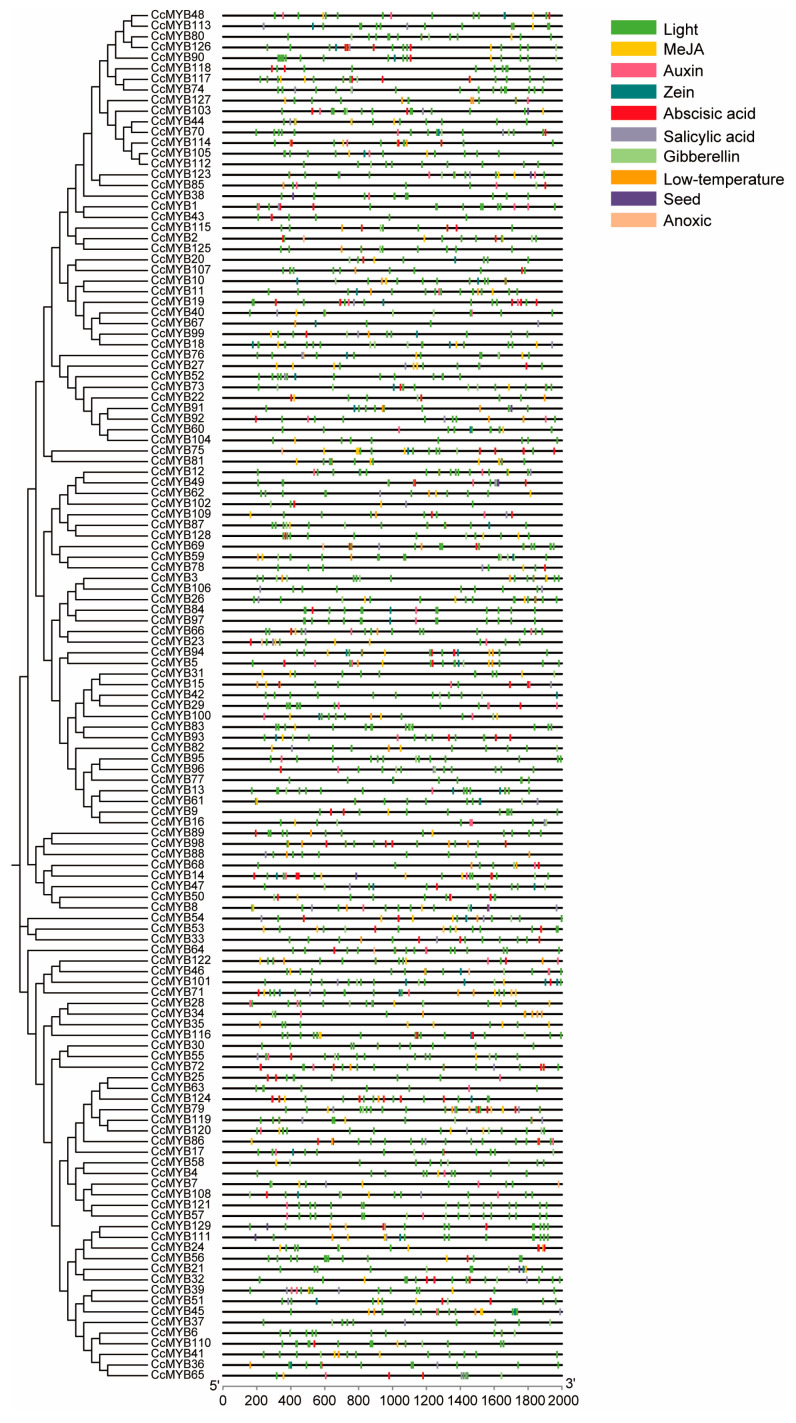
Analysis of cis-acting elements in the promoter region of the *CcMYB* genes. A 2 kb sequence of the promoter region of the *CcMYB* genes was extracted and analyzed, and different cis-acting elements are marked with specific colors.

Multiple cis-regulatory elements were identified within the promoter region of the *CcMYB* genes, indicating the presence of a complex regulatory network that governs its expression (Figure 4). Notably, the promoter region contained plant hormone-responsive elements, such as MeJA, ABRE, and SARE, suggesting that the expression of the *CcMYB* genes may be influenced by various phytohormones. Furthermore, stress-related cis-acting elements, including DSRE and LTRE, were also identified, implying that the *CcMYB* genes may be involved in mediating the plant’s response to diverse abiotic stress conditions.

### 3.5. Chromosomal Distribution and Synteny Analysis of CcMYB Genes

**Figure 5:**
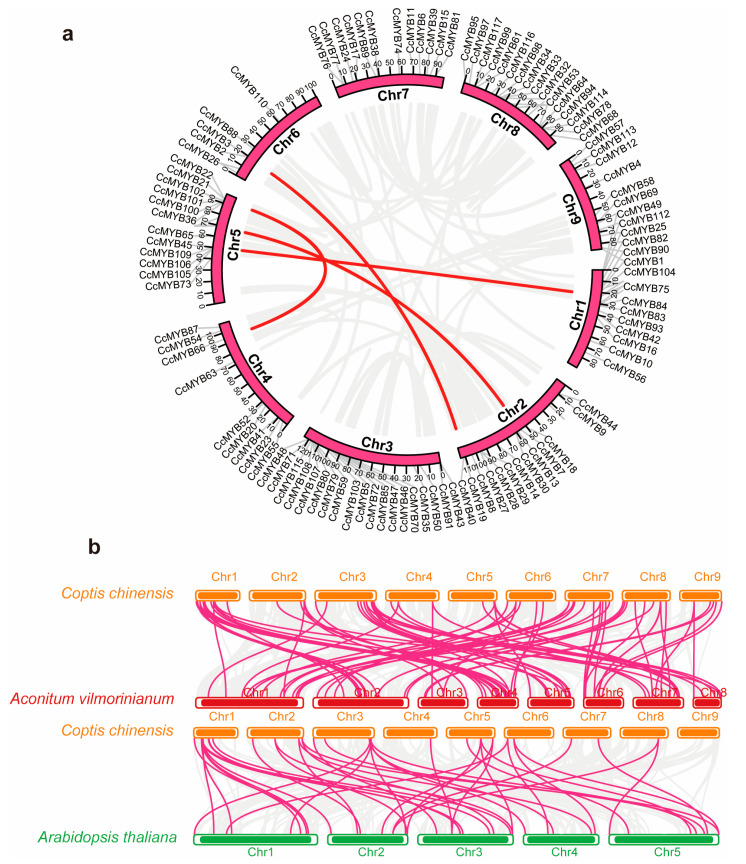
(**a**) Syntenic analysis of *CcMYB* genes in *C. chinensis*. Red lines represent segmental duplication *CcMYB* gene pairs in the genome, and chromosome numbers are labeled next to each chromosome. (**b**) Syntenic analysis of *CcMYB* genes between *C. chinensis* and *A. vilmorinianum* and between *C. chinensis* and *A. thaliana*, where red lines represent *CcMYB* genes with collinearity and blue lines indicate *CcMYB* genes with segmental duplication.

To further elucidate the evolutionary dynamics of the CcMYB gene family, we investigated the chromosomal distribution of these genes and assessed potential gene duplication events. The 129 *CcMYB* genes were unevenly distributed across nine chromosomes, with the highest concentrations (15–19 genes) observed on chromosomes Chr1, Chr3, Chr5, and Chr8. In contrast, Chr6 harbored the fewest *CcMYB* genes, with only six identified (Figure S1). An analysis of gene duplication events revealed that segmental duplication events were observed between chr1 and chr5; chr2 and chr5; chr2 and chr6; and chr4 and chr5, including eight genes (Figure 5a). This finding suggests that these chromosomes played a central role in the evolutionary expansion of the CcMYB gene family in *C. chinensis*. Additionally, the Ka/Ks ratios for four segmental duplication and two tandem duplication CcMYB gene pairs were below 1 (Table S3), suggesting that purifying selection has been the primary force driving the evolutionary conservation of this gene family. This implies that these genes have been under selective pressure to maintain their functional stability over evolutionary time.

To investigate the evolutionary relationships between *C. chinensis*, the closely related species *Aconitum vilmorinianum*, and the distantly related species *A. thaliana*, gene duplication events were analyzed (Figure 5b). The results revealed that *C. chinensis* shares a closer evolutionary relationship with species within the same family. Furthermore, gene duplication events were predominantly concentrated on chromosomes Chr1, Chr2, Chr4, and Chr5, suggesting that these chromosomes may have played an important role in the evolutionary diversification of the CcMYB gene family.

### 3.6. Expression Patterns of CcMYB in Different Tissues

**Figure 6:**
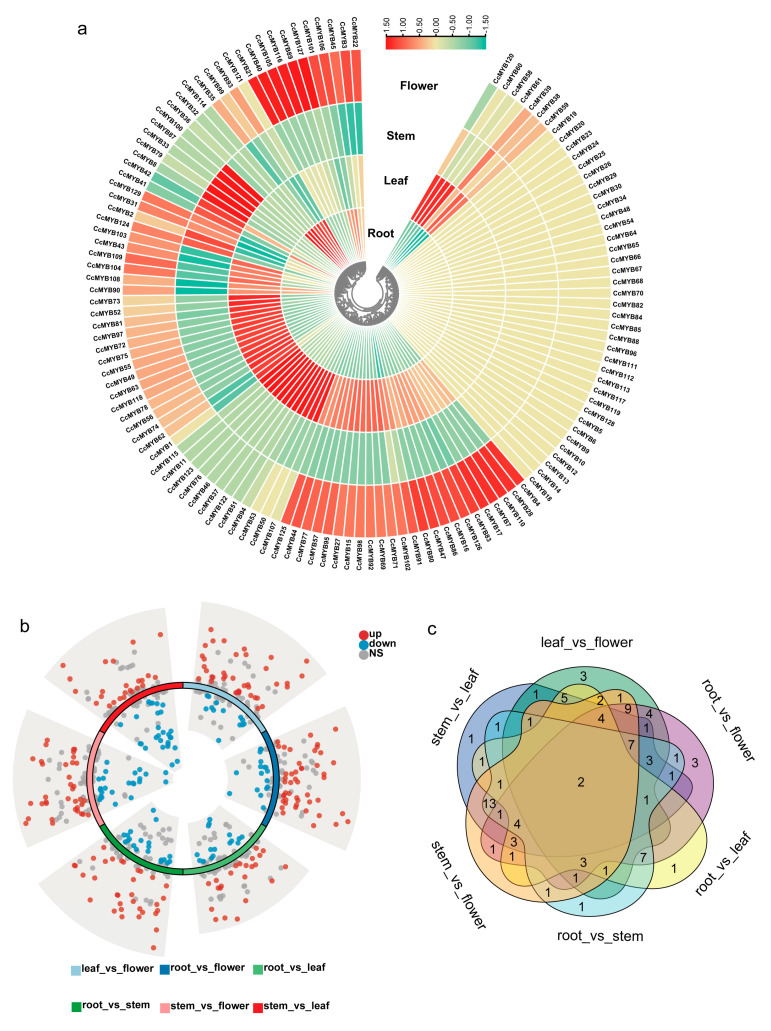
Transcriptome analysis of *CcMYB* genes in different tissues. (**a**) Expression patterns of 129 *CcMYB* genes in four different tissues of *C. chinensis*, where red indicates high expression levels and green indicates lower expression levels. (**b**) Volcano plot of differentially expressed CcMYB genes among different tissues, with red representing upregulated genes and blue representing downregulated genes. (**c**) Venn diagram of differentially expressed *CcMYB* genes among different tissues.

Transcriptomic data revealed the expression levels of *CcMYB* genes across four distinct tissues of *C. chinensis*. A clustered heatmap was generated to visualize the expression profiles of the 129 *CcMYB* genes (Figure 6a). Cluster analysis based on expression patterns showed that different tissues contained subgroups with higher expression levels. Notably, genes such as *CcMYB21*, *CcMYB40*, *CcMYB105*, and *CcMYB116* exhibited high expression exclusively in the stem. Additionally, *CcMYB4*, *CcMYB28*, and *CcMYB110* showed elevated expression levels in the leaf (Table S4). Subsequently, we conducted an in-depth analysis of the differences in *CcMYB* gene expression across various tissues and found that a significant number of *CcMYB* genes exhibited marked differences. In the comparison between the leaf and flower, we identified 37 upregulated *CcMYB* genes and 13 downregulated *CcMYB* genes. The comparison between the root and flower revealed 43 upregulated *CcMYB* genes and 15 downregulated ones. In the comparison of the root and leaf, we found 20 upregulated *CcMYB* genes and 16 downregulated ones. The comparison between the root and stem showed 31 upregulated *CcMYB* genes and 18 downregulated ones. When comparing the stem and flower, we identified 33 upregulated *CcMYB* genes and 22 downregulated genes (Figure 6b). Lastly, in the comparison between the stem and leaf, we found 22 upregulated *CcMYB* genes and 28 downregulated genes. Additionally, we observed significant differences in the expression of *CcMYB109* and *CcMYB17* across different tissues (Figure 6c).

**Figure 7:**
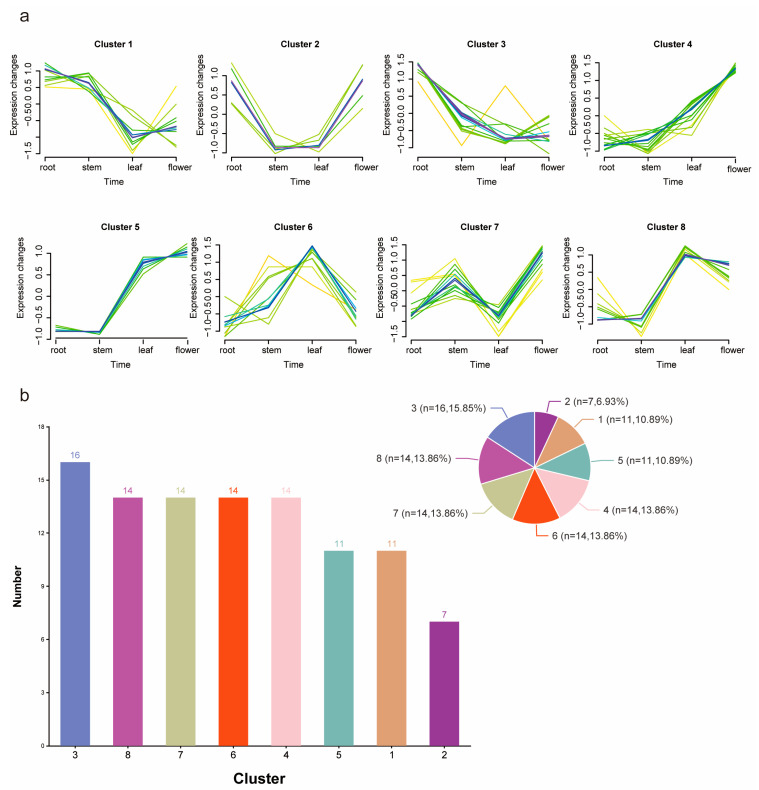
Trend analysis of *CcMYB* genes among different tissues. (**a**) Trend clustering statistics of four tissues: root, stem, leaf, and flower. Different colors represent different *CcMYB*. (**b**) Number of *CcMYB* genes in different trend categories.

To better investigate the expression trends of different *CcMYB* genes across various tissues, we performed clustering analysis based on the expression levels of *CcMYB* genes in different tissues. The results indicated that 129 *CcMYB* genes could be categorized into eight main trends across four distinct tissues (Figure 7a). The largest cluster, Cluster 3, contained the highest number of *CcMYB* genes, totaling 16. Clusters 4, 6, 7, and 8 each contained 14 genes, while Clusters 1 and 5 had 11 genes. The smallest cluster, Cluster 7, contained only seven genes (Figure 7b).

### 3.7. Identification of CcMYB Genes Conferring Resistance to Metal Stress

**Figure 8:**
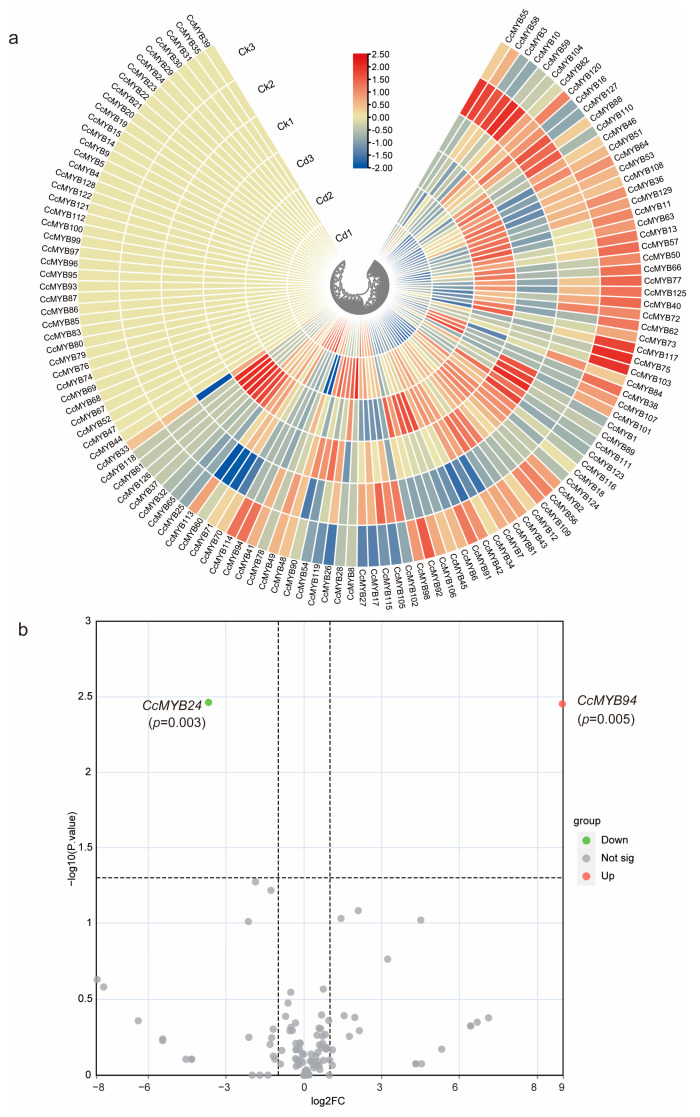
Transcriptome analysis of *CcMYB* genes under control and heavy metal stress conditions. (**a**) Heatmap of the expression levels of *CcMYB* genes in different samples under control and heavy metal stress. (**b**) Volcano plot of differentially expressed *CcMYB* genes.

To investigate the role of *CcMYB* genes in *C. chinensis’s* ability to resist heavy metals, we downloaded transcriptomic data related to heavy metal stress from public databases, with a focus on cadmium stress. The results indicated that the expression levels of most *CcMYB* genes decreased under heavy metal stress (Figure 8a), and only *CcMYB94* was found to be upregulated (Figure 8b). In addition, *CcMYB120* and *CcMYB110* also exhibited increased expression levels under heavy metal stress, although the *p*-value was not statistically significant.

### 3.8. qRT-PCR Analysis

**Figure 9:**
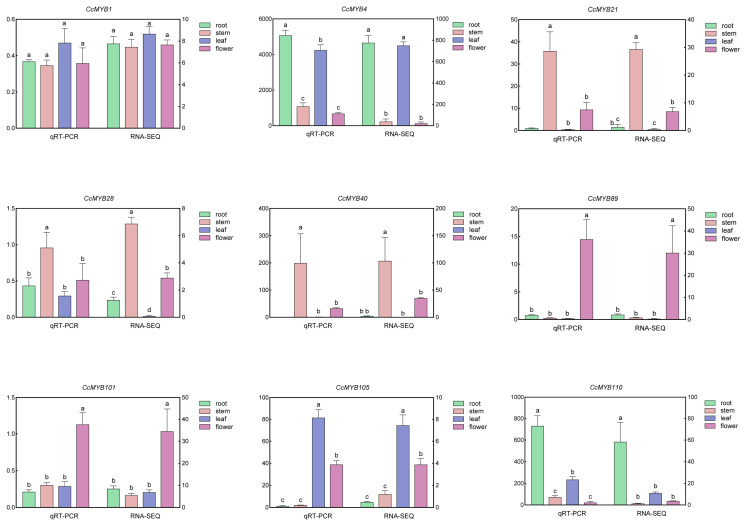
Verification of the expression levels of 9 randomly selected *CcMYB* genes in different tissues using qRT-PCR. (a, b, c, d) indicate significant differences between groups: the same letters indicate no significant difference between groups, while different letters indicate significant differences between groups.

To validate the reliability of the transcriptome results, nine *CcMYB* genes (*CcMYB1*, *CcMYB4*, *CcMYB21*, *CcMYB28*, *CcMYB40*, *CcMYB89*, *CcMYB101*, *CcMYB105*, and *CcMYB110*) were randomly selected for qRT-PCR analysis. The results indicated a strong correlation between the expression levels of these genes and the transcriptome data, further confirming the accuracy of the transcriptome analysis (Figure 9).

## 4. Discussion

The MYB gene family members are crucial components of plant signaling networks. *C. chinensis* is an herbaceous plant widely used in traditional Chinese medicine, with its main active compound, berberine, exhibiting significant biological activity [1]. Moreover, even within the same species, the medicinal properties of *C. chinensis* vary considerably between countries. In recent years, two complete genomes of *C. chinensis* have been successfully sequenced and published [37,38], providing a valuable data foundation for a more comprehensive analysis of its characteristics and a deeper understanding of its medicinal value. As one of the largest transcription factor families in plants, the MYB family plays a key role in regulating various plant functions, including growth, metabolism, and stress responses [8]. MYB transcription factors have been widely reported in many important crops and model plants, such as rice [39], soybean [40], *A. thaliana* [11], maize [41], and potato [42]. This study provides a complete identification and systematic analysis of the MYB transcription factor family in *C. chinensis* based on whole-genome data, contributing to a better understanding of the characteristics of the MYB family in this medicinal plant.

The total number of MYB transcription factors varies significantly across plant species. In this study, we identified 129 MYB genes in *C. chinensis*, fewer than the 168 in the model species *A. thaliana*. Statistical analysis (χ^2^ test) confirmed this difference as significant (*p* < 0.01), potentially reflecting evolutionary divergence in gene family expansion between the two species. Focusing on the well-studied R2R3-MYB subclass, *A. thaliana* contains 126 R2R3-MYB genes, while *C. chinensis* has 83, a difference that is also statistically significant (χ^2^ test, *p* < 0.01). Compared to other Ranunculaceae species, the R2R3-MYB gene count in *C. chinensis* (83) matches that of *C. teeta* (88) [43] with no significant difference (χ^2^ test, *p* > 0.05) but differs significantly from *Aquilegia coerulea* (103; χ^2^ test, *p* < 0.05) [44] and *Kingdonia uniflora* (123; χ^2^ test, *p* < 0.01) [43]. From a classification perspective, the MYB family in *C. chinensis* diverges from other *Ranunculaceae* species. Based on the *A. thaliana* classification system, the R2R3-MYB genes in *C. chinensis* were categorized into 16 subgroups, whereas those in *C. teeta*, *A. coerulea*, and *K. uniflora* were divided into 28, 25, and 25 subgroups, respectively, a variation likely due to species-specific differences. Additionally, phylogenetic analysis identified 19 distinct subfamilies in *C. chinensis*, with the S6 subfamily unique to this species. Further investigation into the functions of genes within the S6 subfamily may hold significant value.

Whole-genome duplication events are common in most angiosperms and are considered key mechanisms for plant adaptation to environmental changes [45]. The expansion of transcription factor families is often linked to whole-genome duplication, with fragment and tandem duplications being the most common forms [46]. To explore the expansion and adaptive evolution of the MYB gene family in *C. chinensis*, we identified MYB genes with duplication events at the gene family level. In *C. chinensis*, we observed four pairs of *CcMYB* genes that underwent fragment duplication, which may play a role in the synthesis of secondary metabolites. To further understand the functional divergence and adaptation of MYB gene duplications, we calculated the Ka/Ks values of the duplicated gene pairs. By analyzing the Ka/Ks values, we can assess the selective pressures during gene expansion, revealing whether the gene family has undergone functional conservation or diversification. The Ka/Ks values of the gene pairs in *C. chinensis* were all below 1, indicating that these genes have undergone purifying selection. Fragment duplication of MYB family members has also been reported in other species, such as the seven species of the genus *Ipomoea* [47], *Theobroma cacao* [48], and *Brassica oleracea* [49]. This indicates that while the number of duplicated *MYB* genes varies among species, this phenomenon is widespread across many species. Similar duplication events are not limited to the MYB family; similar fragment duplications have also been observed in other transcription factor families, such as the NAC family in *Ammopiptanthus mongolicus* [50] and *Castanea mollissima* [51]. These findings suggest that fragment duplication events in transcription factor families are widespread across genomes and play an important role in gene family expansion, functional differentiation, and species adaptation.

*C. chinensis* is a significant medicinal plant, with its primary active constituents comprising flavonoids and alkaloids (e.g., berberine). Extensive studies have demonstrated that the pharmacologically active compounds in plants are often concentrated in their roots, stems, and leaves [52], with alkaloids in *C. chinensis* predominantly accumulating in the stems. Previous research has established that MYB transcription factors play a pivotal role in regulating the flavonoid biosynthesis pathway [53]. In this study, transcriptomic analysis revealed that genes such as *CcMYB21*, *CcMYB40*, *CcMYB105*, and *CcMYB116* exhibit stem-specific high expression in *C. chinensis*. Homology alignment with *A. thaliana* indicated that *CcMYB21* and *CcMYB105* are homologous to MYB genes involved in the bHLH-WD40-MYB ternary complex. To date, no studies have identified MYB transcription factors that directly regulate the berberine biosynthesis pathway in plants. However, evidence from certain species suggests that MYB factors may indirectly participate in alkaloid biosynthesis by cooperating with transcription factors such as WRKY. Given that the flavonoid and alkaloid biosynthesis pathways share common precursors derived from phenylpropanoid metabolism, we hypothesize that these *CcMYB* genes, which exhibit high expression in the stems of *C. chinensis*, may indirectly affect alkaloid biosynthesis in *C. chinensis* by regulating the expression of upstream genes in the phenylpropanoid metabolism pathway.

Heavy metal contamination, especially with cadmium (Cd), lead (Pb), arsenic (As), and chromium (Cr), is one of the major environmental challenges we face today [54]. These metals not only disrupt the plant life cycle but also reduce crop yields and may even lead to plant death [55]. In response, plants have evolved a series of defense mechanisms to combat heavy metal stress. MYB transcription factors in plants are key regulators of stress responses, working in concert with downstream target genes to mitigate the harmful effects of heavy metals. For example, the overexpression of *BpMYB1* in *B. papyrifera* enhances its ability to accumulate and tolerate Cd [56]. In *Raphanus sativus*, the overexpression of *RsMYB1* increases anthocyanin accumulation and enhances heavy metal stress tolerance in transgenic *R. sativus* [57]. In *Boehmeria nivea*, the overexpression of *BnMYB2* increases Cd tolerance and accumulation in transgenic *A. thaliana* plants [58]. Moreover, the R2R3-MYB transcription factor *MYB49* has been reported to regulate cadmium accumulation [59]. In this study, using transcriptomic data from cadmium stress, we analyzed the differentially expressed MYB genes in *C. chinensis*. Except for *CcMYB94*, which was upregulated, the other *CcMYB* genes were downregulated, suggesting that MYB genes can respond to cadmium stress, though the specific regulatory mechanisms still require further investigation.

The findings of this study provide a foundation for understanding the MYB transcription factor family in *C. chinensis*, particularly its roles in the biosynthesis of secondary metabolites and responses to heavy metal stress. Currently, research on the biosynthetic pathways and regulatory mechanisms of natural active compounds remains limited, with few target genes identified as regulators of metabolic pathways. Future studies could prioritize functional validation of key *CcMYB* genes potentially involved in the synthesis of medicinal compounds in *C. chinensis*, such as *CcMYB21*, *CcMYB40*, *CcMYB105*, and *CcMYB116*, using techniques like CRISPR/Cas9-mediated gene editing or overexpression in model plants to clarify their precise roles in regulating flavonoid and alkaloid biosynthesis. Ultimately, investigating the biosynthesis of pharmacologically active compounds in medicinal plants and their gene regulatory patterns will facilitate the application of genetic engineering approaches to enhance plant secondary metabolic pathways.

## 5. Conclusions

In this study, we identified a total of 129 CcMYBs in *C. chinensis*. We predicted their physicochemical properties and subcellular localization, with the results indicating that most CcMYBs are situated in the nucleus. Gene structure analysis and conserved motif analysis revealed the conservation and specificity of CcMYBs. The analysis of cis-regulatory elements identified a rich diversity of cis-regulatory elements in CcMYBs, suggesting that they may be involved in hormone regulation and stress response. Phylogenetic analysis indicated that CcMYBs comprise 19 subfamilies, with S6 being unique, which implies that CcMYBs may have diverse biological functions. Synteny analysis showed that phylogenetic duplication played a role in the expansion of the CcMYB gene family. Transcript level analysis conducted on different tissues, along with quantitative real-time PCR (qRT-PCR) analysis, revealed unique expression patterns of *CcMYB* genes and their role in heavy metal stress resistance, suggesting their importance in biosynthesis and responses to abiotic stress. In summary, this study provides a foundational understanding of MYB transcription factors in *C. chinensis* and offers new insights into their roles in alkaloid biosynthesis and regulation of responses to heavy metal stress. These analyses lay the groundwork for future research on the regulatory mechanisms of plant secondary metabolism and stress resistance.

## Data Availability

In this study, the *C. chinensis* genome data were downloaded from the NCBI database (PRJNA662860); the transcriptome data from the roots, stems, leaves, and flowers of *C. chinensis* were obtained from the SRA database (PRJNA649082); and the transcriptome data under cadmium stress were downloaded from the China National Genomics Data Center (CNCB), with the accession number CRA013690.

## Supplementary Materials

The following supporting information can be downloaded at https://www.mdpi.com/article/10.3390/genes16050476/s1, Figure S1: Chromosomal localization analysis of CcMYB. Chromosome numbers are labeled on the left side of each chromosome. The scale is in megabases (Mb); Table S1: qRT-PCR primers for *CcMYB* genes; Table S2: Statistical analysis of the physicochemical properties of CcMYBs; Table S3: Evolutionary analysis of *CcMYB* gene selection; Table S4: Transcriptome FPKM data of *CcMYB* genes for the roots, stems, leaves, and flowers in *C. chinensis.*
