# Supplementary figures and images for: Genome-Wide Screening of the MYB Genes in Coptis chinensis and Their Roles in Growth, Development, and Heavy Metal Resistance
Source: Genes (Basel). 2025 Apr 23;16(5):476. doi: 10.3390/genes16050476 (PMC12111318; doi:10.3390/genes16050476)

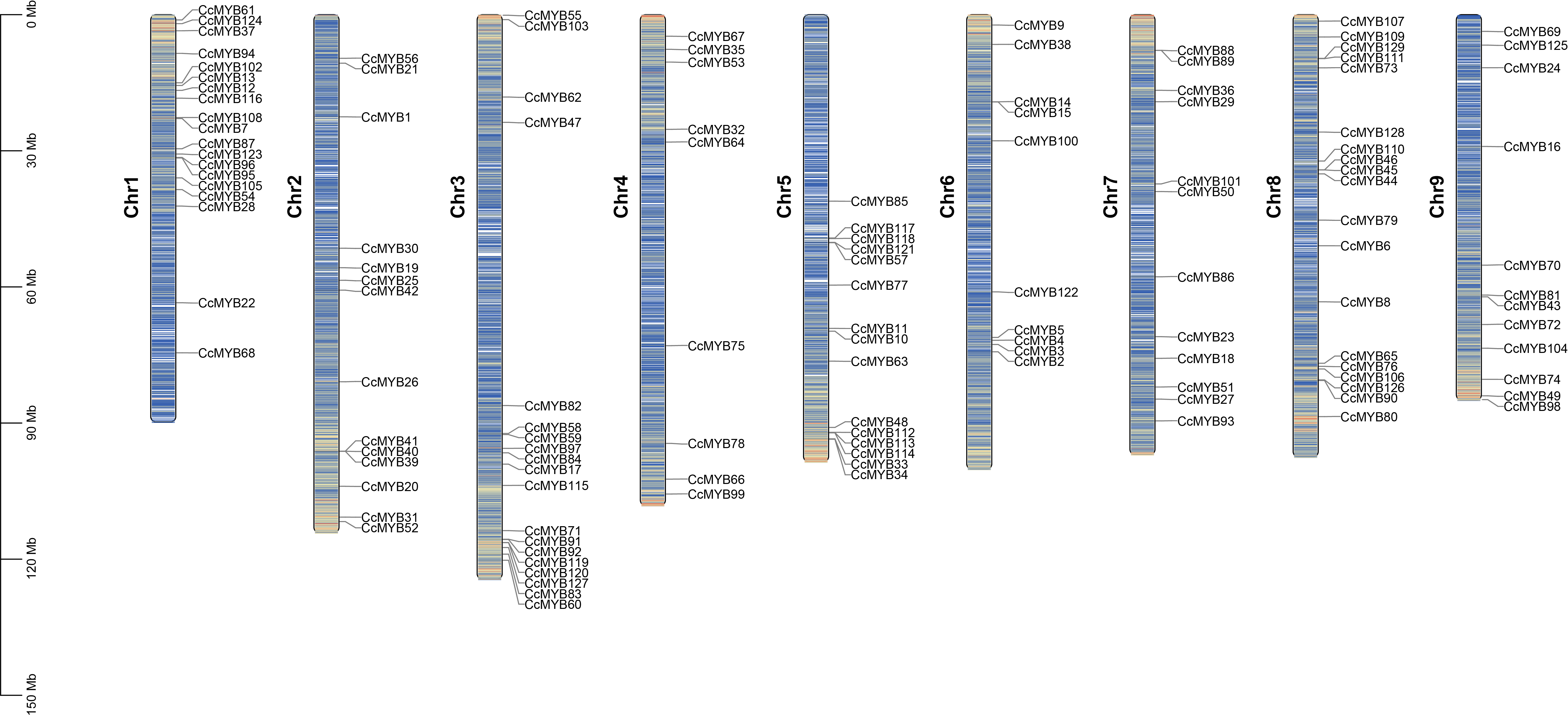

Supplement: Supplementary file 1 [file genes-16-00476-s001.zip › Figure S1.tif]
